# The Effect of Quality of Life on Medication Compliance Among Dialysis Patients

**DOI:** 10.3389/fphar.2018.00488

**Published:** 2018-06-05

**Authors:** Hiroyuki Nagasawa, Tomoya Tachi, Ikuto Sugita, Hiroki Esaki, Aki Yoshida, Yuta Kanematsu, Yoshihiro Noguchi, Yukio Kobayashi, Etsuko Ichikawa, Teruo Tsuchiya, Hitomi Teramachi

**Affiliations:** ^1^Department of Pharmacy, Secomedic Hospital, Funabashi, Japan; ^2^Laboratory of Clinical Pharmacy, Gifu Pharmaceutical University, Gifu, Japan; ^3^Department of Pharmacy, Chiba Central Medical Center, Chiba, Japan; ^4^Department of Pharmacy, Chuno Kosei Hospital, Gifu, Japan; ^5^Community Health Support and Research Center, Gifu, Japan; ^6^Laboratory of Community Healthcare Pharmacy, Gifu Pharmaceutical University, Gifu, Japan

**Keywords:** dialysis, patient, quality of life, medication compliance, multiple logistic regression analysis

## Abstract

Dialysis treatment is known to lead to reduced quality of life (QOL) among patients. This decreased QOL is believed to influence medication compliance, although this effect has not yet been clarified. In this study, we investigated whether decreased QOL due to dialysis treatment does in fact influence medication compliance. Participants were 92 patients who self-managed their medication and were receiving dialysis treatment at Secomedic Hospital or Chiba Central Medical Center. We surveyed their age, sex, dialysis period, and medication management situation, and administered the EQ-5D and Kidney Disease Quality of Life Instrument–Short Form. A multiple logistic regression analysis with medication compliance as the dependent variable and QOL as the independent variable was conducted. The recovery rate and effective response rate were both 100%. The results indicated that patients with good sleep QOL (mean or above) had higher odds of medication compliance (odds ratio, 3.36; 95% confidence interval, 1.26–8.96; *P* = 0.016). Therefore, improving the quality of sleep of dialysis patients might help to improve their medication compliance.

## Introduction

Globally, an estimated 1.4 million patients received renal replacement therapy, including dialysis treatment, in 2001 (World Health Organization, 2017). In the U.S., approximately 680,000 patients are reported to have undergone dialysis or received a kidney transplant at the end of 2014 (United States Renal Data System, 2017). In Japan, the number of dialysis patients per 1 million individuals was 1,830 in 2001, which is the highest in the world (Moeller et al., 2002), and the number of dialysis patients exceeded 300,000 in 2011 (The Japanese Society for Dialysis Therapy, 2017).

Most patients with chronic kidney failure are treated with dialysis (either hemodialysis or peritoneal dialysis). In recent years, through the continued development of medical technology, materials, and medications for dialysis treatment, medical professionals have begun to emphasize both prolonging patients' lives and maintaining and improving their quality of life (QOL), including their activities of daily living, health, role functioning, and social functioning. As dialysis treatment generally involves visiting the hospital two to three times per week for upwards of around 3 h each time, it is believed to have a large effect on patients' QOL. To date, research on dialysis patients has indicated that some aspects of QOL are lowered in dialysis patients (Perlman et al., 2005; Kalender et al., 2007; Mazairac et al., 2012; Erez et al., 2016).

As secondary symptoms of the underlying disease of kidney failure frequently are exhibited during the introduction, maintenance, and terminal periods of dialysis (Chiu et al., 2009; Tessari et al., 2009; Li et al., 2013), many dialysis patients undergo multidrug therapy. The complexity of multidrug therapy in dialysis patients makes them aware of the high risk of adverse events, which leads to subsequent non-compliance. Medication non-compliance averts patients from gaining the full benefit of the prescribed medications and is associated with increased mortality and hospitalizations (Saran et al., 2003; Denhaerynck et al., 2007). Therefore, compliance with medication therapy is a key component of the effective management of dialysis patients. A number of studies have focused on the medication management situation of dialysis patients, particularly their medication adherence (Loghman-Adham, 2003; Karamanidou et al., 2008; Lindberg and Lindberg, 2008; Schmid et al., 2009; Browne and Merighi, 2010; Neri et al., 2011; Rosenthal Asher et al., 2012; García-Llana et al., 2013; Chan et al., 2014; Van Camp et al., 2014; Burnier et al., 2015; Ghimire et al., 2015; Wileman et al., 2015; Freire de Medeiros et al., 2017; Jalal et al., 2017; Tohme et al., 2017). At present, however, the relationship between the QOL of dialysis patients and their oral medication management status has not been clarified. Understanding this is extremely important for providing appropriate treatment and care for such patients. Accordingly, in this study, a survey of dialysis patients was conducted, with the objective of clarifying the effect of QOL of dialysis patients on medication compliance.

## Methods

### Study design

This cross-sectional survey study was implemented using a self-administered questionnaire. Completion of the questionnaire took approximately 10 min and was thus quick and easy for responders to complete.

### Participants and implementation period

Of the patients who received dialysis treatment at Secomedic Hospital and Chiba Central Medical Center in Japan between June 1, 2015 and December 31, 2015, 92 patients who self-managed their medication were selected as study participants.

### Health-related quality of life instruments

Patient background information included age, sex, dialysis period, disease causing hemodialysis, and comorbidities. The comorbidities were classified according to International Statistical Classification of Diseases and Related Health Problems (ICD-10) (World Health Organization, 2016^1^). These data were extracted from patients' electronic medical charts.

To assess patient QOL, we used the EuroQol 5-dimension questionnaire (EQ-5D) (Nishimura et al., 1998) for general QOL, and the Kidney Disease Quality of Life Instrument Short Form version 1.3 (KDQOL-SF) (Green et al., 2001) for kidney disease-specific QOL. Registration of use of EQ-5D and KDQOL-SF was performed prior to study implementation. EQ-5D and SF-36 are used in numerous countries. For SF-36, national standard values (national norms) are published for each country, enabling determinations as to whether a QOL score is higher or lower than the relevant national standard values.

The EQ-5D is a QOL survey that comprises a 5-item scale and a visual analog scale (VAS). It is widely used in clinical research and to examine the health status of the general population as a comprehensive scale for cardinally evaluating changes in health status (Nishimura et al., 1998). For the 5-item scale, health conditions are classified into five areas of “mobility,” “self-care,” “usual activities,” “pain/discomfort,” and “anxiety/depression,” and participants are asked to rate each on three levels: “no problems” (Level 1), “some problems” (Level 2), and “problems” (Level 3). A utility value is calculated by combining the five item scores and converting this sum using the Japanese version of a utility value conversion table. The VAS utilizes a 20-cm line ranging from 0 (“the worst health condition imaginable”) to 100 (“the best health condition imaginable”) (Nishimura et al., 1998).

The KDQOL-SF comprises a kidney disease-specific scale, a non-health-related QOL scale, and a comprehensive QOL scale (Fukuhara et al., 1998a,b). The kidney disease-specific scale comprises 40 items divided into eight subscales: “symptoms,” “effect of kidney disease,” “burden of kidney disease,” “work status,” “cognitive function,” “social interaction,” “sexual function,” and “sleep.” The non-health-related QOL scale comprises four items divided into the three subscales: “social support,” “dialysis staff encouragement,” and “patient satisfaction.” For both, scoring is done at the subscale level, with a minimum value of 0 and a maximum value of 100. Higher scores indicate higher QOL. The comprehensive QOL scale is used to assess health-related QOL, and is composed of 36 items divided into eight subscales: “physical functioning,” “role physical,” “bodily pain,” “general health,” “vitality,” “social functioning,” “role emotional,” and “mental health.” Using the subscale scores, it is possible to calculate a norm-based scoring (NBS) score based on the national standard and three summary scores [“physical component summary” (PCS), “mental component summary” (MCS), and “role-social component summary” (RCS)]. Note that the NBS score and summary scores are displayed as deviation scores using a mean of 50 and a standard deviation of 10 as the national standard value.

### Medication compliance tool

**Figure 1:**
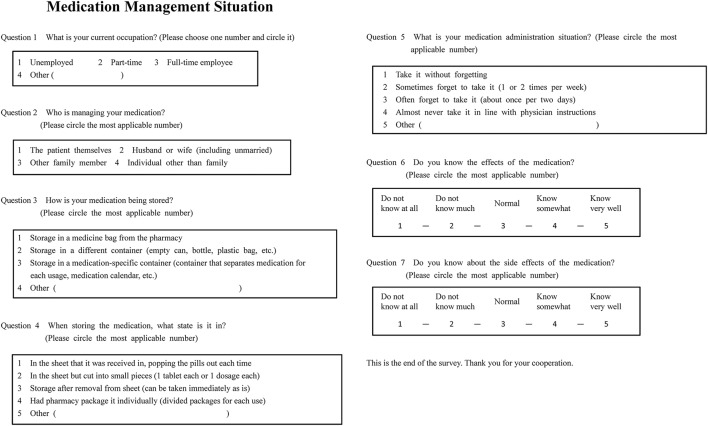
Survey regarding medication management situation.

Medication management situation was investigated using an original questionnaire form (Figure 1). This form contained items of “occupation,” “medication management,” “medication storage,” “medication storage state,” “medication administration situation,” “knowledge of effects,” and “knowledge of side effects.” The number of medications being taken and the frequency of administration were extracted from patients' electronic medical charts.

In our study, we evaluated medication compliance (whether patients took prescribed medicines or not) and not medication adherence. As a tool to evaluate medication compliance, we used an original self-reported questionnaire, “Medication Administration Situation” (Question No. 5 in Figure 1). The question was “Shat is your medication administration situation?” and the answer choices were “take it without forgetting,” “sometimes forget to take it (1 or 2 times per week),” “often forget to take it (about once per 2 days), “almost never take it in line with physician instructions,” or “other.”

### Stratifications

**Figure 2:**
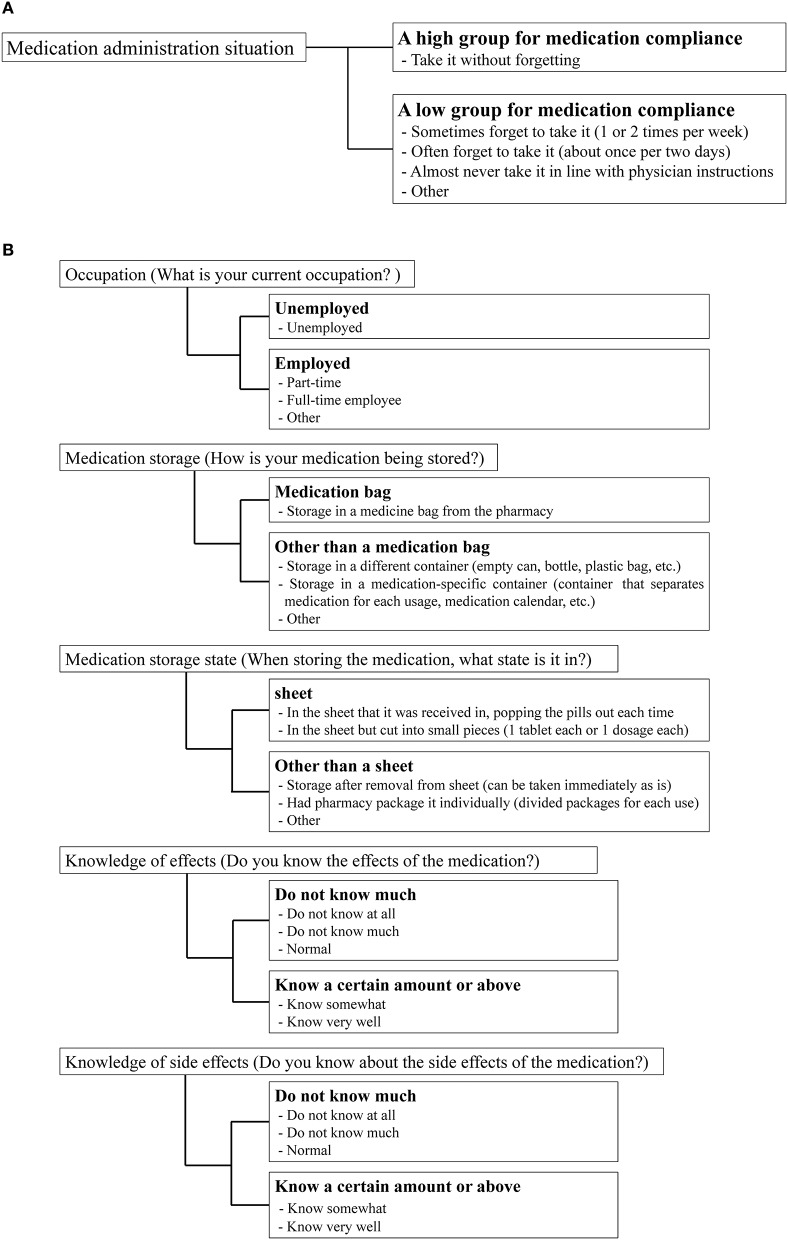
Stratification criteria for medication compliance and medication management situation. **(A)** Medication compliance. **(B)** Medication management situation.

To investigate the effect of each variable on medication compliance, we performed the following stratifications. Participants were stratified into groups of high and low medication compliance based on their responses to the medication administration situation item (Figure 2A). Specifically, individuals who responded that they “take it without forgetting” were classified into the high medication compliance group, while those who answered that they “sometimes forget to take it (1 or 2 times per week),” “often forget to take it (about once per 2 days), “almost never take it in line with physician instructions,” or “other” were classified into the low medication compliance group. We also stratified patient characteristics, EQ-5D scores, KDQOL-SF scores, number of medications taken, and frequency of administration according to the criteria in Table 1. The standard utility value was set as the utility value obtained by Fujikawa et al. (0.877) for members of the general population (Fujikawa et al., 2011). Medication management situations aside from “medication administration situation” were stratified using the criteria in Figure 2B.

**Table 1:** Stratification criteria.

**Characteristics**		
Age (years)	≥65	< 65
Sex	Male	Female
Dialysis period (months)	≥Median	< Median
Causative disease	Diabetes mellitus	Kidney disease
QOL		
EQ-5D		
Utility value (EQ-5D)	≥Standard value	< Standard value
KDQOL-SF		
Kidney disease-specific scale		
Symptoms/Problems	≥Mean	<Mean
Effects of kidney disease	≥Mean	<Mean
Burden of kidney disease	≥Mean	<Mean
Work status	≥Mean	<Mean
Cognitive function	≥Mean	<Mean
Quality of social interaction	≥Mean	<Mean
Non-health related QOL scale		
Social support	≥Mean	<Mean
Dialysis staff encouragement	≥Mean	<Mean
Patient satisfaction	≥Mean	<Mean
Summary scores		
PCS	≥50	<50
MCS	≥50	<50
RCS	≥50	<50
Number of medications	≥7	<7
Frequency of administration	≥6	<6
(times/day)		

*QOL, Quality of life; PCS, Physical component summary; MCS, Mental component summary; RCS, Role-social component summary*.

### Statistical analysis

The univariate analysis was performed with Fisher's exact test to investigate the differences in patients' medication management situation (excluding “medication administration situation” and “medication management”), QOL, and characteristics between the high and low medication compliance groups. For the multivariate analysis, we used multiple logistic regression analysis, with “medication administration situation” as the dependent variable and characteristics, EQ-5D utility value, kidney disease-specific scale scores, non-health-related QOL scale scores, summary scores, the medication management situation items (excluding “medication administration situation” and “medication management”) with *P* < 0.20 in the univariate analysis as independent variables. A *P* < 0.05 was set as the level of significance. All statistical analyses were conducted using IBM SPSS 24.0J (IBM Corp., Armonk, New York).

### Ethical considerations

This study was conducted in compliance with the Declaration of Helsinki and Ethical Guidelines for Research Involving Human Subjects after receiving approval from the ethical review board of Gifu Pharmaceutical University (Approval No.: H27-13), Secomedic Hospital (Approval No.: SM2015-27-2), and Chiba Central Medical Center (Approval No.: H27-K2). This survey was conducted after thoroughly explaining it to participants via documents and obtaining their written consent.

## Results

### Recovery rate and effective response rate

The questionnaire recovery rate was 100% (92/92), as was the effective response rate.

### Patient characteristics

Descriptive statistics for patient characteristics are indicated in Table 2. Participants' mean (± standard deviation was 67.0 ± 11.6 years, 21 were female (22.8%), and the median dialysis period was 81.5 months. Causative diseases are diabetes mellitus (50.0%) and kidney disease (50.0%).

**Table 2:** Patient characteristics.

	***n* = 92**
Age (years)	
Average ± standard deviation	67.0 ± 11.6
Sex	*n* (%)
Male	71 (77.2)
Female	21 (22.8)
Dialysis period (months)	
Median value	81.5
(interquartile range)	(29–136.5)
Causative disease	
Diabetes mellitus	46 (50.0)
Kidney disease	46 (50.0)
Comorbidities (ICD-10)	
1. Certain infectious and parasitic diseases	12 (13.0)
2. Neoplasms	7 (7.6)
3. Diseases of the blood and blood-forming organs and certain disorders involving the immune mechanism	31 (33.7)
4. Endocrine, nutritional, and metabolic diseases	88 (95.7)
5. Mental and behavioral disorders	11 (12.0)
6. Diseases of the nervous system	18 (19.6)
7. Diseases of the eye and adnexa	51 (55.4)
8. Diseases of the ear and mastoid process	3 (3.3)
9. Diseases of the circulatory system	90 (97.8)
10. Diseases of the respiratory system	24 (26.1)
11. Diseases of the digestive system	57 (62.0)
12. Diseases of the skin and subcutaneous tissue	15 (16.3)
13. Diseases of the musculoskeletal system and connective tissue	54 (58.7)
14. Diseases of the genitourinary system	9 (9.8)
15. Pregnancy, childbirth, and the puerperium	0 (0.0)
16. Certain conditions originating in the perinatal period	0 (0.0)
17. Congenital malformations, deformations, and chromosomal abnormalities	1 (1.1)
18. Symptoms, signs, and abnormal clinical and laboratory findings, not elsewhere classified	34 (37.0)
19. Injury, poisoning, and certain other consequences of external causes	7 (7.6)
20. External causes of morbidity and mortality	0 (0.0)
21. Factors influencing health status and contact with health services	0 (0.0)
22. Codes for special purposes	0 (0.0)
Education		
Elementary or junior high graduate	26 (28.3)
High school graduate or university entrance exam	40 (43.5)
Technical school graduate or university drop-out	12 (13.0)
Junior college graduate	0 (0.0)
University graduate (4 years or more)	14 (15.2)
Graduate school completed	0 (0.0)
Married		
Yes	73 (79.3)
No	19 (20.7)
Housemates		
Lives alone	18 (19.6)
Husband or wife (including unmarried)	43 (46.7)
Other family members	31 (33.7)
Individual other than a family member	0 (0.0)
Questionnaire completion period		
During dialysis treatment	74 (80.4)
After bringing home	18 (19.6)
Other	0 (0.0)
Assistance completing questionnaire		
Yes	76 (82.6)
No	16 (17.4)
Reason for assistance (*n* = 76)		
Vision impaired	7 (9.2)
Unable to write	69 (90.8)
Other	0 (0.0)

### Medication management situation

The results for medication management situation are indicated in Table 3. Regarding the most common responses, for the occupation item, 66 participants (71.7%) indicated “unemployed”; for medication storage, 40 participants (43.5%) indicated “storage in a different container (empty can, bottle, plastic bag, etc.)”; for medication storage state, 36 participants (39.1%) indicated “in the sheet but cut into small pieces (1 tablet each or 1 dosage each)”; and for medication administration situation, 63 participants (68.5%) indicated “take it without forgetting.” As for knowledge of effects, 19 participants (20.7%) indicated that they “know very well” and 33 individuals (35.9%) indicated that they “know somewhat.” Meanwhile, for side effects, only 5 participants (5.4%) indicated “know very well” and 15 (16.3%) indicated “know somewhat.” The mean number of medications taken was 9.8 ± 3.8, and the frequency of administration in 1 day was 5.6 ± 2.2 times.

**Table 3:** Survey results (medication management situation).

	***n* = 92**
	*n* (%)
Occupation		
Unemployed	66 (71.7)
Part-time	3 (3.3)
Full-time employee	21 (22.8)
Other	2 (2.2)
Medication storage		
Storage in a medicine bag from the pharmacy	28 (30.4)
Storage in a different container	40 (43.5)
(empty can, bottle, plastic bag, etc.)	
Storage in a medication-specific container (container separating medication by usage, medication calendar, etc.)	24 (26.1)
Other	0 (0.0)
Medication storage state		
In the sheet as it was received, popping the pills out each time	22 (23.9)
In the sheet but cut into small pieces (1 tablet each or 1 dosage each)	36 (39.1)
Removed from sheet (unwrapped pills)	3 (3.3)
Packaged individually by pharmacy	31 (33.7)
Other	0 (0.0)
Medication administration situation		
Take it without forgetting	63 (68.5)
Sometimes forget to take it (1 or 2 times per week)	25 (27.2)
Often forget to take it (about once per two days)	3 (3.3)
Almost never take it in line with physician instructions	1 (1.1)
Other	0 (0.0)
Knowledge of effects		
Do not know at all	10 (10.9)
Do not know much	19 (20.7)
Normal	11 (12.0)
Know somewhat	33 (35.9)
Know very well	19 (20.7)
Knowledge of side effects		
Do not know at all	34 (37.0)
Do not know much	24 (26.1)
Normal	14 (15.2)
Know somewhat	15 (16.3)
Know very well	5 (5.4)
	**Mean** ± **Standard deviation**
Number of medications	9.8 ± 3.8
Frequency of administration (times/day)	5.6 ± 2.2

### QOL evaluation

The EQ-5D results are indicated in Table 4. The item with the highest response rate of “no problems” (level 1) was “physical functioning” with 88 participants (95.7%). Conversely, the item with the lowest response rate for “no problems” was “pain/discomfort” with 54 individuals (58.7%). The mean utility value and VAS score were 0.809 ± 0.184 and 66.6 ± 18.3, respectively.

**Table 4:** Patient QOL (EQ-5D).

	***n* = 92**
	*n* (%)
Mobility	
No problems	61 (66.3)
Problems	30 (32.6)
Confined to bed	1 (1.1)
Self-care	
No problems	88 (95.7)
Problems	4 (4.3)
Unable	0 (0.0)
Usual activities	
No problems	62 (67.4)
Problems	28 (30.4)
Unable	2 (2.2)
Pain/discomfort	
None	54 (58.7)
Moderate	32 (34.8)
Extreme	6 (6.5)
Anxiety/depression	
None	73 (79.3)
Moderate	17 (18.5)
Extreme	2 (2.2)
	**Mean** ± **Standard deviation**
Utility value	0.809 ± 0.184
Health condition	66.6 ± 18.3

*EQ-5D, EuroQol 5-dimension*.

Table 5 indicates the results of the KDQOL-SF. On the kidney-disease-specific scale and the non-health-related QOL scale, the mean scores for “social interaction” (95.9 ± 8.3) and “cognitive function” (94.1 ± 11.8) were high, while that for “burden of kidney disease” (47.4 ± 25.3) was rather low. We did not conduct an analysis for “sexual function,” as there was considerable missing data for this variable (due to a refusal to answer). As for the comprehensive QOL scale, “vitality” (53.0 ± 12.2) and “mental health” (55.9 ± 9.9) scores were higher than the national standard value, while “physical functioning” (37.4 ± 25.3), “role physical” (37.5 ± 20.6), “general health,” (44.3 ± 11.1), and “role emotional” (46.8 ± 16.7) were lower than the national standard. As for the summary scores, the MCS score (58.8 ± 9.9) was higher than the national standard value, while the PCS (34.8 ± 15.9) and RCS (47.7 ± 16.0) scores were below it.

**Table 5:** Patient QOL (KDQOL-SF).

	***n* = 92**
	**Mean** ± **Standard deviation**
Kidney disease-specific scale	
Symptoms	87.0 ± 9.7
Effects of kidney disease	85.2 ± 10.1
Burden of kidney disease	47.4 ± 25.3
Work status	58.7 ± 22.9
Cognitive function	94.1 ± 11.8
Quality of social interaction	95.9 ± 8.3
Sexual function	Not determined
Sleep	74.0 ± 20.4
Non-health related QOL scale	
Social support	80.4 ± 19.8
Dialysis staff encouragement	83.3 ± 17.0
Patient satisfaction	87.0 ± 12.5
Comprehensive QOL scale (NBS score)	
Physical functioning	37.4 ± 19.7
Role physical	37.5 ± 20.6
Bodily pain	51.1 ± 13.4
General health	44.3 ± 11.1
Vitality	53.0 ± 12.2
Social functioning	51.5 ± 10.9
Role emotional	46.8 ± 16.7
Mental health	55.9 ± 9.9
Summary Scores	
PCS	34.8 ± 15.9
MCS	58.8 ± 9.9
RCS	47.7 ± 16.0

*QOL, Quality of life; KDQOL-SF, The Kidney Disease Quality of Life instrument Short Form version1.3; NBS, Norm-based scoring; PCS, Physical component summary; MCS, Mental component summary; RCS, Role-social component summary*.

### Results of univariate analysis

The results of the univariate analysis of patient characteristics and QOL in the high and low medication compliance groups are indicated in Table 6. In the high medication compliance group, the percentage of patients with above average sleep scores was significantly higher than that in the low compliance group (*P* = 0.043).

**Table 6:** Univariate analysis results.

	**Medication compliance**		
	**Low group (*****n*** = **29)**	**High group (*****n*** = **63)**	***P***
	***n*** **(%)**	***n*** **(%)**	
Characteristics			
Age (≥65 years)	17 (58.6)	43 (68.3)	0.480
Sex (female)	6 (20.7)	15 (23.8)	0.796
Dialysis period (≥median)	14 (48.3)	33 (52.4)	0.823
Causative disease (kidney disease)	11 (37.9)	35 (55.6)	0.178
EQ-5D			
Utility value (≥standard value)	9 (31.0)	29 (46.0)	0.254
KDQOL-SF			
Kidney disease-specific scale			
Symptoms (≥mean)	15 (51.7)	40 (63.5)	0.361
Effect of kidney disease (≥mean)	15 (51.7)	34 (54.0)	1.000
Burden of kidney disease (≥mean)	18 (62.1)	29 (46.0)	0.182
Work status (≥mean)	7 (24.1)	12 (19.0)	0.589
Cognitive function (≥mean)	21 (72.4)	52 (82.5)	0.280
Quality of social interaction (≥mean)	20 (69.0)	46 (73.0)	0.804
Sleep (≥mean)	11 (37.9)	39 (61.9)	0.043^*^
Non-health related QOL scale			
Social support (≥mean)	20 (69.0)	33 (52.4)	0.175
Dialysis staff encouragement (≥mean)	19 (65.5)	31 (49.2)	0.179
Patient satisfaction (≥mean)	14 (48.3)	21 (33.3)	0.248
Summary score			
PCS (≥50)	4 (13.8)	13 (20.6)	0.568
MCS (≥50)	25 (86.2)	51 (81.0)	1.000
RCS (≥50)	16 (55.2)	38 (60.3)	0.656
Medication management situation			
Occupation (employed)	17 (58.6)	42 (66.7)	0.489
Medication storage (medication bag)	8 (27.6)	20 (31.7)	0.809
Medication storage state (sheet)	18 (62.1)	40 (63.5)	1.000
Knowledge of effects (Know a certain amount or above)	18 (62.1)	34 (54.0)	0.505
Knowledge of side effects (Know a certain amount or above)	7 (24.1)	13 (20.6)	0.787
Number of types of medication (≥7)	23 (79.3)	53 (84.1)	0.567
Frequency of administration (≥6 times/day)	13 (44.8)	37 (58.7)	0.262

* *P < 0.05. QOL, Quality of life; EQ-5D, EuroQol 5 dimension; PCS, Physical component summary; MCS, Mental component summary; RCS, Role-social component summary*.

### Results of multivariate analysis

**Figure 3:**
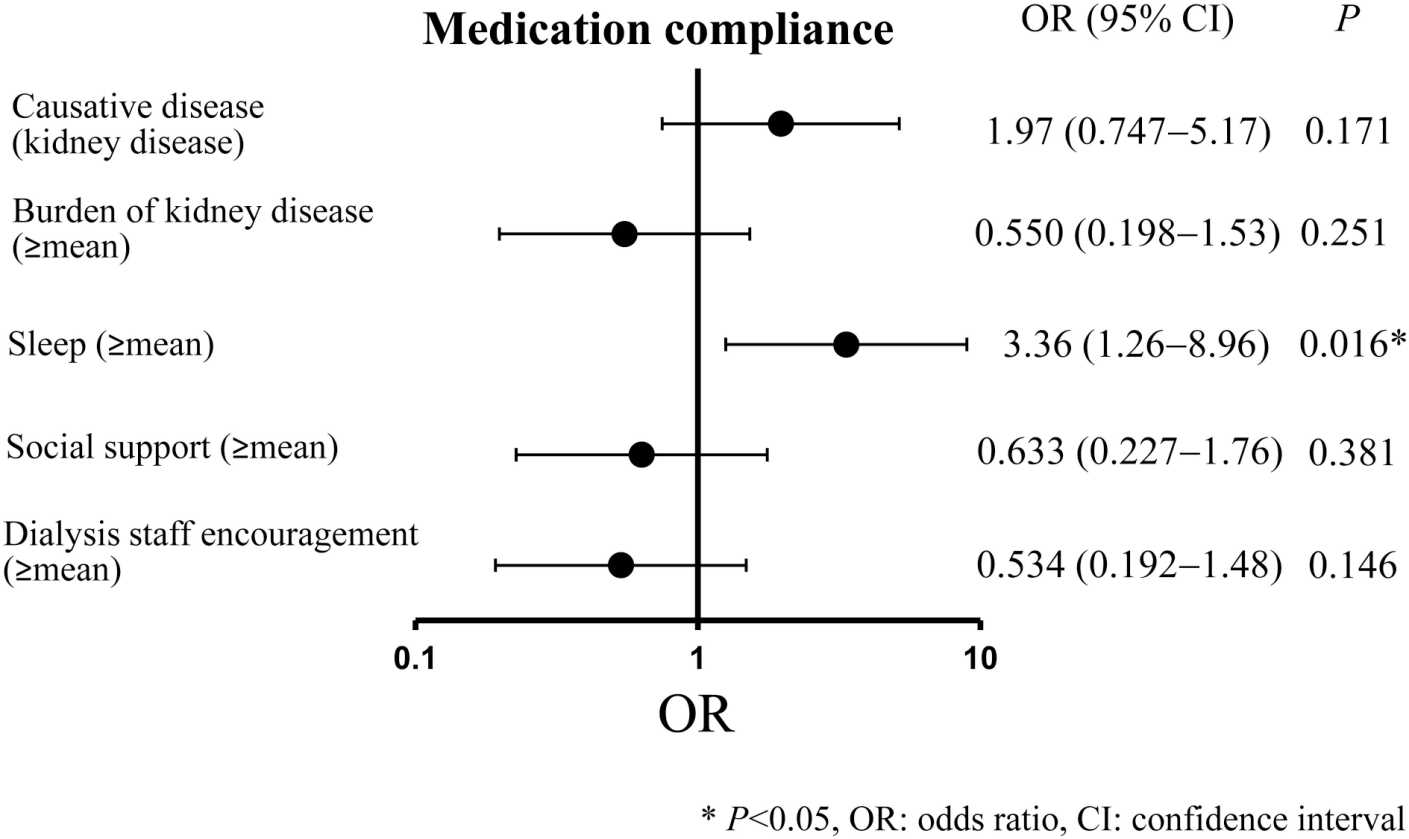
Multivariate analysis results. ^*^P < 0.05, OR: odds ratio, CI: confidence interval.

The results of the multiple logistic analysis conducted with “medication compliance” as the dependent variable and “causative disease,” “burden of kidney disease,” “sleep,” “social support,” and “dialysis staff encouragement” as independent variables (as they all had a *P* < 0.20 in the univariate analysis) are indicated in Figure 3. A significant association was observed for sleep (≥mean) (odds ratio, 3.36; 95% confidence interval, 1.26–8.96; *P* = 0.016).

## Discussion

The response rate of questionnaire in the patients in our study was high, which would be owing to the fact that patients could fill the questionnaire during dialysis (about 3).

The results of the medication management situation indicate dialysis patients who use creative methods such as switching containers or cutting the medication sheet, while approximately 1 in 3 had low medication compliance. Further, while over half of patients knew about the effects of the medications, comparatively few patients knew about the side effects. From the results of the mean number of medications being taken and the mean frequency of administration in 1 day, the daily number of medications taken, and the frequency of administration appeared to be high among dialysis patients in this study. Because dialysis patients have lowered kidney function, professionals generally believe it best to avoid polypharmacy. However, polypharmacy is often necessary to manage the many secondary symptoms of the disease causing the kidney failure during dialysis treatment (Chiu et al., 2009; Tessari et al., 2009; Li et al., 2013; St Peter, 2015). The same was found in the present study. Medication compliance is important for hemodialysis patients because many hemodialysis patients are non-adherent to medication therapy. It has been reported that the rates of non-adherence to oral medications in chronic hemodialysis patients ranged from 3 to 80% (Schmid et al., 2009) and that approximately half of hemodialysis patients are non-adherent to medication therapy (Neri et al., 2011). However, non-adherence rates have been reported to be lower in Japan than in the U.S. (Miyata et al., 2017). The same was found in the present study. The results would be due to many hemodialysis patients who use creative methods such as switching containers or cutting the medication sheet, which were recommended by pharmacists.

Dialysis patients have been found to show decreases in some areas of QOL compared to healthy individuals (Yoshiya et al., 2001; Erez et al., 2016; Raspovic et al., 2017). In response to evaluation of QOL using the EQ-5D, most individuals indicated that they have no problems in “physical functioning,” whereas comparatively fewer participants had no problems with “pain/discomfort.” Thus, many dialysis patients retain their physical functioning, many of who appear to have pain or discomfort. The utility index value we obtained was close to the value (0.754) (Katayama et al., 2014) obtained by Katayama et al. but lower than the value (0.877) (Fujikawa et al., 2011) obtained in the survey of the general population by Fujikawa et al.

On the kidney-disease-specific scale and the non-health-related QOL scale of the KDQOL-SF, scores for “cognitive function” and “social interaction” were high, while that for “burden of kidney disease” was low. On the comprehensive QOL scale, the scores of “vitality,” “mental health,” and “MCS” were higher than the national standard value, while the score for “physical functioning,” “role physical,” “general health,” “role emotional,” “PCS,” and “RCS” were lower. Similar to our study, past QOL research on dialysis patients in foreign countries using the kidney-disease-specific scale revealed that the “burden of kidney disease” scores were low (Mazairac et al., 2012; Erez et al., 2016). Furthermore, compared to a control group, scores for all items on the 36-item Short Form Health Survey (which makes up the comprehensive QOL scale in this study) were significantly lower among patients receiving dialysis (Perlman et al., 2005; Kalender et al., 2007). Our findings are consistent with previous studies that have similarly reported that patients with kidney disease have scores below the national standards for PCS, but scores close to national norms for patient MCS (Mazairac et al., 2012; Erez et al., 2016). However, prior research has reported that many dialysis patients suffer depression. Depression constitutes a portion of the mental-health QOL items (depending on extent of depressive symptoms) (Palmer et al., 2013), but does not necessarily entail low patient MCS. While prior reports have shown values for patient MCS that are close to the national norm (Mazairac et al., 2012; Erez et al., 2016), in our study, patient MCS was higher which may be a finding specific to the Japanese sample.

As for the multivariate analysis, we found, when compared to patients with low sleep-related QOL, that patients with high sleep-related QOL had significantly better medication compliance. One potential reason for this is that patients with high sleep QOL tend to be living properly regulated lives, which means that they are perhaps more likely to properly regulate their medication administration as well. Previous studies reported, among patients with schizophrenia, that a decrease in quality of sleep was related to a decrease in adherence (Afonso et al., 2014). The same was found among HIV-positive patients (Saberi et al., 2011). Therefore, improving quality of sleep might help to improve medication compliance for dialysis patients as well.

Factors associated with non-adherence in hemodialysis patients have been reported, including socio-demographic variables such as age and gender; clinical variables such as long-term on hemodialysis and comorbidity; psycho-social variables such as depressive symptoms and belief about medicine; medication-related factors such as knowledge about medicine and numbers of prescribed medicines (Ghimire et al., 2015). In our study, age, gender, dialysis period, causative disease, numbers of prescribed medicines were not found to be factors associated with medication non-compliance. However, the limitations of this study include the fact that all the dialysis patients we analyzed were on hemodialysis and that none were on peritoneal dialysis. The study sample was small and limited to a specific locality. The high response rate might represent the population with effective medication compliance and influence our study results. The method to measure medication compliance was self-reported questionnaires but not direct methods including pill count and use of electronic monitoring devices. Furthermore, there might be unmeasured confounding factors in the multivariate analysis. Further large studies might be required to reach a robust conclusion.

## Conclusion

This study revealed that high sleep-related QOL is associated with better medication compliance. Therefore, utilizing dialysis treatment and care that supports patients' lifestyle habits, including sleep, may help improve their medication compliance.

## Author contributions

All authors contributed to the study design. All authors participated in collecting and interpreting the data. HN, TT, and IS analyzed data and drafted the manuscript. TT confirmed the analyzed data and revised the manuscript. All authors reviewed and approved the final manuscript.

### Conflict of interest statement

The authors declare that the research was conducted in the absence of any commercial or financial relationships that could be construed as a potential conflict of interest.

## Footnotes

^1^World Health Organization (2016). Available online at: http://apps.who.int/classifications/icd10/browse/2016/en
